# Body-weight-specific and shared metabolomic responses to acute sleep loss in young adults

**DOI:** 10.1186/s12967-026-08350-4

**Published:** 2026-06-03

**Authors:** Linnéa Good, Luiz Eduardo Mateus Brandão, Lieve Thecla van Egmond, Payam Emami Khoonsari, Ida Erngren, Henrik Carlsson, Robert Fredriksson, Kim Kultima, Christian Benedict, Jonathan Cedernaes

**Affiliations:** 1 Department of Medical Cell Biology, Uppsala University, Uppsala, Sweden; 2 Department of Medical Sciences, Uppsala University, Akademiska sjukhuset, Ing. 40, 5 tr, Uppsala, Sweden; 3 Department of Psychiatry and Psychotherapy, Tübingen Center for Mental Health (TüCMH), University of Tübingen, Tübingen, Germany; 4 German Center for Mental Health (DZPG) Partner Site Tübingen, Tübingen, Germany; 5 Department of Biochemistry and Biophysics, National Bioinformatics Infrastructure Sweden, Science for Life Laboratory, Stockholm University, Stockholm, Sweden; 6 Department of Pharmaceutical Biosciences, Uppsala University, Uppsala, Sweden

**Keywords:** Acylcarnitines, Body weight category, Kynurenine pathway, Overnight wakefulness, Sex-specific metabolism, Simulated shift work

## Abstract

**Introduction:**

Insufficient sleep increases the risk of weight gain and metabolic dysfunction, while obesity is linked to disrupted sleep and metabolic impairment. Although both conditions are tightly interconnected, it remains unclear whether body weight status determines how acute sleep loss alters metabolite levels. We aimed to assess whether acute sleep loss elicits different metabolic responses in individuals with and without obesity, and whether these responses are modified by sex.

**Methods:**

In a within-subject, randomized crossover study, 42 adults (mean age 24.9 years; 18 women, 24 men; 18 with obesity; 24 with normal weight) completed one night of total sleep deprivation (TSD) and one night of normal sleep (NS; 8 h). Morning fasted blood samples were analyzed using targeted metabolomics. Mixed-effects models were used to assess within-individual changes from NS to TSD, and to determine the magnitude and direction of these changes within each body weight group and sex.

**Results:**

In response to TSD compared with NS, tryptophan levels increased (+12%, FDR < 0.05) only in participants with obesity, while histidine, L-kynurenine, and creatinine levels decreased (4–21%, FDR < 0.05) only in normal-weight individuals. Notably, the kynurenine-to-tryptophan ratio decreased after TSD in both groups, and levels of circulating medium-chain acylcarnitines (+26–39%) increased within both weight groups – together indicating shared metabolic responses to acute sleep deprivation. Exploratory sensitivity analyses indicated that several of these responses were sex-specific. Polysomnography-based sleep architecture during NS, available in a subset of 36 participants, was largely comparable between body weight groups, suggesting that observed metabolic differences were not attributable to baseline differences in sleep structure.

**Conclusions:**

Acute sleep loss elicits both shared and body-weight-specific changes in circulating metabolites in young adults. These findings indicate that body-weight status shapes which metabolic pathways respond to a single night of sleep loss, and in what direction, but larger studies are required to confirm the body weight- and sex-specific findings. Given that acute sleep loss is a common metabolic stressor that increases cardiometabolic risk, these differences may contribute to mechanistic hypotheses about the heightened vulnerability to metabolic dysregulation in individuals with obesity who are frequently exposed to sleep loss.

**Supplementary information:**

The online version contains supplementary material available at 10.1186/s12967-026-08350-4.

## Introduction

Insufficient sleep constitutes a risk factor for adverse cardiometabolic effects, such as weight gain and obesity [1]. Conversely, obesity is also associated with disrupted sleep and worse sleep quality [2, 3]. This may contribute to impaired metabolic integrity and disrupted energy homeostasis, as these processes are directly impacted by sleep. Epidemiological studies indicate that variation in sleep duration may result in differential effects on cardiometabolic outcomes in individuals with versus without obesity [4]. However, we currently lack experimental studies to disentangle the mechanistic relationships.

In studies examining healthy individuals, curtailed sleep has been found to adversely impact the regulation of several metabolic hormones, such as the levels of ghrelin, leptin and adiponectin, as well as glucometabolic pathways [5–10]. These changes may mechanistically explain how disrupted sleep, for example, promotes overeating and insulin resistance, at least in normal-weight individuals.

We previously reported that individuals with obesity have higher circulating levels of orexigenic ghrelin in response to experimental sleep loss. In contrast, only normal-weight individuals exhibit increased adiponectin levels following a night of sleep loss, compared with after a normal night of sleep [11]. However, whether the within-subject metabolic response to acute sleep loss differs in individuals with obesity and normal-weight individuals, remains to be elucidated.

Several features of obesity could plausibly modify how acute sleep loss perturbs systemic metabolism. Obesity is characterized by reduced metabolic flexibility, altered mitochondrial fatty-acid handling, low-grade inflammation, and dysregulation of HPA-axis and sympathetic responses to acute stressors [12–15]. Each of these systems is also engaged by acute sleep loss. Whether obesity therefore amplifies or reshapes the metabolic response to sleep loss differently compared with individuals with normal weight has not, to our knowledge, been examined within a single crossover comparison.

Circulating levels of certain metabolites, such as acylcarnitines, are altered by sleep loss, possibly providing a mechanistic link for how sleep loss can worsen insulin resistance [8, 9]. Acylcarnitines have also been associated with sleep duration and variability in both normal weight and overweight or obesity [16, 17]. Establishing if such metabolites respond differently depending on body weight may provide insight into whether the long term risk of chronic sleep loss or shift work differs for individuals with and without obesity.

Building on this background, we used metabolomics to explore whether body weight status shapes the metabolomic response to one night of sleep loss, compared with one full night of sleep. The crossover design of the study, in which each participant serves as their own control across both conditions, isolated the acute effect of sleep loss from the inter-individual metabolic variability that characterizes obesity. As additional exploratory secondary analyses, we also examined within each weight group whether the responses to acute sleep loss differed by biological sex.

## Methods

### Participants

The data comes from a subsample of a larger study investigating the health effects of sleep and sleep loss on several health outcomes [11]. For the present analysis, 42 participants from this larger study had available metabolomics data, which have not been published elsewhere. Eighteen of these participants were classified as having obesity (8 females, age 24.8 ± 0.8 years, BMI 34.1 ± 0.8 kg/m^2^), based on the definition below, and the rest were classified as having a normal body weight (10 females, age 25.0 ± 0.5 years, BMI 22.4 ± 0.4 kg/m^2^). Sample size was based on similar prior studies in the field of sleep and untargeted biomarker analyses [8, 18]; therefore, no power calculation was done.

Obesity was defined as having a BMI of ≥30 kg/m^2^, and a waist circumference measuring > 102 cm for males and >88 cm for females [19]. Normal weight was defined as having a BMI of <25 kg/m^2^ and a waist circumference of <94 cm for males and <88 cm for females.

All participants were screened to ensure they were generally healthy and had no somatic or psychiatric diseases. In addition, no shift workers or people who travelled across time zones in the last month were included in the study. All women who participated were on combined hormonal contraceptives. The study was conducted at the Biomedical center at Uppsala university, Sweden, in accordance with the Declaration of Helsinki and was approved by the ethical board of Uppsala, Sweden (Dnr 2017/560). All participants provided oral and written informed consent prior to the study and were compensated for participation. The ethical board of Uppsala did not classify the study to be a clinical trial. Therefore, the clinical trial was not preregistered.

### Study design and procedure

Using a randomized crossover setup, all participants underwent two sessions, i.e., one night of total sleep deprivation (TSD), and one night of normal sleep (NS) with an 8-hour sleep opportunity. An in-lab adaptation night preceded (within 7 days) each participant’s first session. The order of the sessions was counterbalanced across participants, and there was a washout period of one week in between the sessions. All women’s sessions were scheduled during the active phase of hormonal contraceptive treatment.

Participants were continuously supervised during the in-lab sessions, starting with a standardized meal at 7:00 pm. During the NS sessions, sleep was scheduled from 11:00 pm to 7:00 am Sleep duration and architecture was determined from a polysomnography recording (SOMNO HD, SOMNOmedics GmbH). During the overnight TSD sessions, participants did low-demanding sedentary activities such as watching movies, reading books, or playing board games in standard indoor lighting (~500 lux).

In both the NS and TSD sessions, blood was collected at 7:30 am after an overnight fast and centrifuged at 1300 relative centrifugal force (RCF) for 10 minutes at 4 °C. The plasma supernatant was then aliquoted and stored at −80 °C until further analysis.

### Sample preparation

Plasma samples (50 µL) were thawed and protein-precipitated with 150 µL ice-cold methanol containing isotopically labeled internal standards (IS). The samples were vortexed for 15 seconds, then precipitated at −20 °C for 60 min, and subsequently centrifuged at 21,100 RCF for 15 min at 4 °C. The supernatants were stored at −80 °C prior to analysis.

Samples were prepared in batches consisting of 36 samples, including two blank samples, one with IS and one without, and one external quality control sample (QC). Blank samples were prepared with water instead of plasma. A pooled QC sample was prepared by pooling the study samples.

### HPLC-HRMS analysis

Samples were analyzed in a constrained randomized order, with samples from the same individual analyzed consecutively. QC samples and Blank samples were analyzed at set intervals throughout the analysis. The HPLC-MS system used was an Ultimate 3000 HPLC system (Thermo Scientific, Waltham, MA, USA) interfaced with a high-resolution hybrid quadrupole Q Exactive Orbitrap MS (Thermo Scientific).

Two µL of each sample was separated on an Accucore 150 Amide column (100 × 2.1 mm, 2.6 µm, Thermo Scientific), using an 18.5 min elution gradient. The gradient was programmed as follows: 0% A for 2.5 min, 0–100% A for 8.75 min, hold at 100% A for 2 min, return to 0% A over 0.25 min, followed by re-equilibration at 0% A for 5 min. Mobile phase A consisted of 60% acetonitrile, 40% H_2_O, 10 mM ammonium formate, pH 3, and Mobile phase B consisted of 95% acetonitrile, 5% H_2_O, 10 mM ammonium formate, pH 3. The flow rate was set to 0.6 mL/min throughout the run, except for the re-equilibration, when the flow rate was increased to 1.0 mL/min. The column temperature was 45 °C.

The mass spectrometer was operated in full-scan mode over a m/z range of 55–820, with a resolution of 70,000. Metabolites were detected in positive ionization mode using the following ion-source parameters: spray voltage was 3.5 kV, the S-lens RF level was 50, and the capillary temperature was 320 °C. The sheath gas flow, auxiliary gas flow, and sweep gas flow were 55, 15, and 3, respectively, and the auxiliary gas heater was 450 °C.

### Mass spectrometry data processing

All mass spectrometry data was processed using TraceFinder (v.4.0, Thermo Scientific). The analysis targeted 55 compounds identified with authentic standards, and the compounds were selected within 30 seconds of the expected retention time and a mass error of < 10 ppm.

### EEG analysis

For the EEG dataset, 43 participants were initially included, but 7 were excluded due to failed sleep EEG registration. However, accelerometer measurements and morning feedback from participants confirmed that the seven excluded participants slept for approximately seven or more hours in the sleep condition. Sixteen of the participants from the EEG dataset were classified as having obesity (9 females, age: 25.3 ± 0.9 years, BMI: 33.7 ± 0.8 kg/m^2^) and the remaining participants were classified as having normal weight (9 females, age: 25.2 ± 0.6 years, BMI: 22.6 ± 0.4 kg/m^2^).

Sleep staging was automated using U-Sleep, a fully convolutional deep neural network [20]. Sleep stages were classified for every 30-second epoch throughout the night in accordance with AASM guidelines (i.e., Wake, NREM1, NREM2, NREM3, and REM). The analysis utilized eight electrode derivations from the right and left hemispheres in central (C3–A2, C4–A1), frontal (F3–A2, F4–A1), frontopolar (Fp1-A2, Fp2-A1), and occipital (O1–A2, O2–A1) regions, combined with data from electrooculogram electrodes (EOGr-A1, EOGl-A2).

Before sleep staging, EEG data was pre-processed. First, data was trimmed according to participants’ in-lab sleep opportunity and filtered using a finite impulse response (FIR) filter from the MNE-Python package [21]. This ensured the data length matched participants’ respective bed/wake time within the sleep opportunity window and retained frequency components between 0.3 Hz and 35 Hz. Then, data quality across all channels was visually inspected to identify deviating patterns that could indicate artifacts, noise, or other irregularities, which could impact our analyses. The final hypnogram of each participant was derived by summing the prediction scores from all available EEG-EOG combinations, with the highest accumulated score determining the sleep stage (wake, or sleep stages N1, N2, N3, or REM) for each epoch.

### Statistical analysis

Only metabolites and metabolomics features that were identified in at least 80% of the study participants were used in the analysis. In total, 55 metabolites were targeted and used in the statistical analyses.

All metabolite values were first log2-transformed, and a LOWESS curve normalization was used to account for the sample run order. Linear mixed-effect models were used to determine the effect of TSD compared with NS on each metabolite, using subject as a random effect. To account for multiple comparisons, *p*-values were adjusted using the Benjamini-Hochberg method, and a false discovery rate (FDR) <0.05 was considered significant. The linear mixed models were run separately on the participants with obesity and normal weight, as well as separately on male and females within each of the weight groups. The kynurenine-tryptophan ratio was analyzed using the Wilcoxon signed-rank test for paired data, and a *p*-value < 0.05 was considered significant.

The EEG-based sleep parameters were analyzed using multiple linear regression to determine the effect of weight class on parameter values, adding age and biological sex into the model as covariates.

## Results

### Effect of overnight sleep loss on targeted metabolite levels

**Fig. 1:**
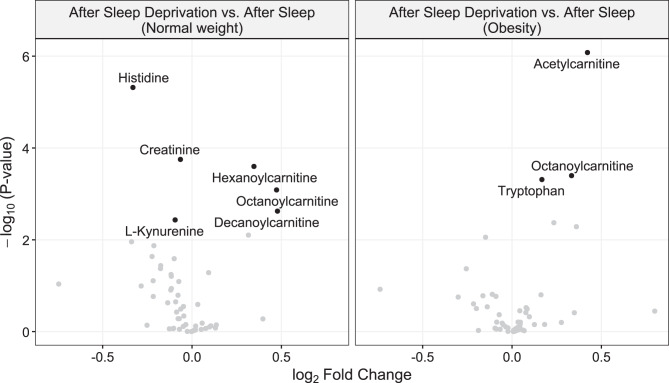
Effect of overnight total sleep deprivation (TSD) compared with a full night of normal sleep (NS) on relative circulating metabolite levels in individuals with normal weight and obesity. A positive coefficient (x axis) indicates higher metabolite levels after TSD compared with NS. Metabolites highlighted in black are significantly altered in the TSD vs. NS condition (FDR < 0.05). Normal weight: *n* = 24; obesity: *n* = 18. Within-subject analyses using mixed modeling

Among medium-chain acylcarnitines, in participants with obesity, compared with NS, we found that one night of sleep loss (TSD) resulted in higher levels of acetylcarnitine (+34%) and octanoylcarnitine (+26%) (both FDR < 0.05). In participants with normal weight, TSD compared with NS resulted in higher levels of hexanoylcarnitine (+27%), octanoylcarnitine (+39%), and decanoylcarnitine (39%) were observed (all FDR < 0.05; Fig. 1). Though most of the altered acylcarnitine metabolites reached FDR < 0.05 only in either the participants with obesity or the participants with normal weight, at FDR < 0.1, all of the aforementioned acylcarnitines were altered by TSD across both weight groups.

Among amino-acid-related metabolites, in participants with obesity, TSD compared with NS resulted in higher levels of tryptophan (+12%), whereas in participants with normal weight, we observed reduced levels of histidine (−21%), creatinine (−4%) and L-kynurenine (−6%) (all FDR < 0.05; Fig. 1). To examine pathway-level dynamics, we calculated the kynurenine-to-tryptophan ratio (KTR) for each individual. We found that the KTR was significantly reduced after TSD compared with NS across both body weight groups (*p* < 0.05; Fig. S1A), indicating that despite exhibiting divergent individual metabolite responses, the response at the pathway level following TSD was directionally consistent across body-weight groups.

**Fig. 2:**
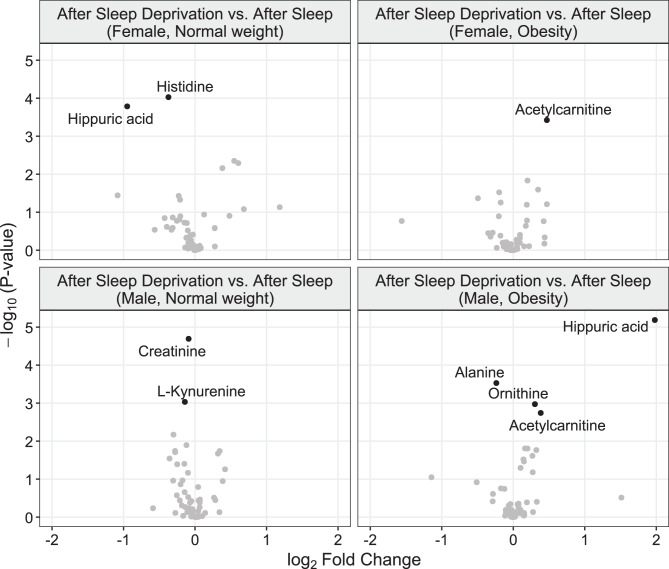
Effect of overnight total sleep deprivation (TSD) compared with a full night of normal sleep (NS) on relative circulating metabolite levels in male and female subjects with normal weight and with obesity. A positive coefficient (x axis) indicates higher metabolite levels after TSD compared with NS. Metabolites highlighted in black are significantly altered in the TSD vs. NS condition (FDR < 0.05). Normal weight: *n* = 10 females and *n* = 14 males, obesity: *n* = 8 females and *n* = 10 males. Analyses done using mixed modeling

We also conducted exploratory sensitivity analyses, to investigate whether the response to TSD was sex dependent. Following TSD compared with NS, levels of acetylcarnitine were increased (+39%) in female participants with obesity, whereas in male participants with obesity, levels of hippuric acid (+294%), ornithine (+23%), and acetylcarnitine (+30%) increased, and levels of alanine (−15%) decreased (all FDR < 0.05). Among participants with normal weight, levels of histidine (−23%) and hippuric acid (−48%) were decreased in females in response to TSD, while in males, levels of creatinine (−6%) and L-kynurenine (−9%) were lower (all FDR < 0.05) (Fig. 2). In sex-specific analyses of the KTR, the ratio was significantly reduced only in men, both with and without obesity (Fig. S1B).

**Fig. 3:**
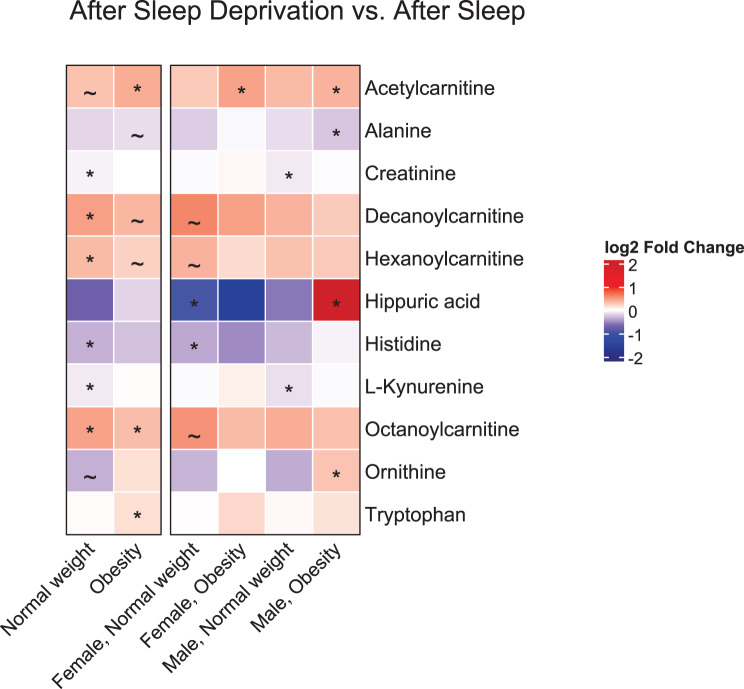
Comparison across body weight categories and sexes for the effects of total sleep deprivation (TSD) compared with a full night of normal sleep (NS) on plasma metabolite levels, for metabolites that were significantly altered in at least one of the analyzed groups. Relative metabolite levels in female and male subjects with normal weight (*n* = 10 and *n* = 14, respectively) and obesity (*n* = 8 and *n* = 10, respectively). A positive coefficient indicates higher metabolite levels after TSD compared with NS, in the mixed modeling analyses. ‘*’ indicates that the metabolite is significantly altered in the TSD vs. NS condition (FDR < 0.05), ‘~’ indicates FDR < 0.1

When examining the direction of change for the significantly altered metabolites in the different groups of subjects in response to TSD compared with NS, most of the metabolites increased or decreased to a similar extent across all the different groups (Fig. 3). Notably, hippuric acid levels were significantly increased in male participants with obesity in response to TSD compared with NS, but the opposite response was seen in female participants with normal weight. Among the metabolites altered by TSD, ornithine, L-kynurenine, and creatinine exhibited within-group coefficients with different directions of change in subjects with obesity versus normal weight, but only the coefficients for one group reached a significance of FDR < 0.05.

### Polysomnographic sleep architecture analysis indicates similar sleep characteristics

In an analysis of sleep parameters and sleep stages from the normal sleep session, we found that the participants with obesity spent slightly more time in N1 than the participants with normal weight (12.1 ± 1.3 versus 17.2 ± 2.7 minutes, *p* < 0.05). No other differences were observed between the groups (Table 1).

**Table 1:** EEG-based sleep parameters during the normal night of sleep, for subjects in the normal weight group (*n* = 20, 9 females) and the group with obesity(*n* = 16, 9 females)

Sleep parameter	Normal weight (n = 20)		Obesity (n = 16)		p-value
	Mean	SEM	Mean	SEM	
TIB (min)	492.3	5.6	486.8	4.5	0.34
SPT (min)	459.1	5.6	450	7.5	0.34
WASO (min)	30.0	8.5	36.7	8.7	0.51
TST (min)	429.1	8.9	413.3	10.2	0.20
SOL (min)	27.0	4.6	26.5	6.7	0.75
NREM (min)	326.8	6.6	321.5	8.7	0.55
N1 (min)	12.1	1.3	17.2	2.7	**0.045**
N2 (min)	214.2	6.6	208.3	9.4	0.49
N3 (min)	100.5	4.7	96.1	4.8	0.54
REM (min)	102.3	6.2	91.8	5.3	0.20
REM latency (min)	109.0	5.4	126.4	11.2	0.20
% NREM	76.4	1.2	77.8	1.2	0.40
% N1	2.9	0.4	4.3	0.4	0.051
% N2	50.0	1.2	50.2	1.6	0.98
% N3	23.5	1.0	23.3	1.2	0.99
% REM	23.6	1.2	22.1	1.2	0.40
% SE	87.3	1.7	85.0	2.2	0.42

Abbreviations: TIB: time in bed, SPT: sleep period time, WASO: wake after sleep onset, TST: total sleep time, SOL: sleep onset latency, NREM: non-rapid eye movement sleep, N1: sleep stage 1, N2: sleep stage 2, N3: sleep stage 3, REM: rapid eye movement sleep

## Discussion

Here, using a randomized within-subject crossover design, we compared the metabolomic response to one night of total sleep deprivation in adults with and without obesity. To our knowledge, this is the first within-subject crossover study to examine how body-weight status may modulate the metabolomic response to acute sleep loss. We identified metabolite levels that were significantly altered only within adults with obesity, as well as metabolites that were significantly altered only within adults without obesity. These findings provide initial mechanistic insight into how body-weight status may shape the metabolic consequences of sleep disruption, and may help explain the heightened metabolic vulnerability associated with obesity.

We found that overnight sleep loss resulted in higher levels of several medium-chain acylcarnitine species across both body weight groups. In line with these results, plasma acylcarnitine levels have been shown to increase after sleep deprivation in multiple studies [8, 9]. Elevated levels of plasma acylcarnitines have been found in individuals with obesity, insulin resistance, and newly diagnosed patients with T2D compared with healthy controls [22–24]. Elevations in our study spanned C2 through C10 medium-chain species across both weight groups, with FDR-corrected significance reached for different individual species in each group but consistent directionality throughout all examined acylcarnitine species. This points to a shared acute response, in which fatty-acid delivery transiently exceeds mitochondrial oxidative throughput in response to sleep loss. It remains an outstanding question whether circulating medium-chain acylcarnitines mechanistically contribute to insulin resistance induced by sleep loss, or if they simply mark an upstream mitochondrial perturbation that drives both phenomena. Distinguishing these possibilities would require studies coupling metabolomics with measures of insulin sensitivity and mitochondrial function within the same sleep loss crossover study.

In the participants with normal weight, we observed lower levels of the creatine breakdown product creatinine in response to TSD. Creatine is involved in peripheral energy metabolism but has also has garnered interest for its central nervous system function, since phosphocreatine buffers ATP in skeletal muscle and cerebral high-energy phosphates have been shown to fall during sleep deprivation. Smaller human trials of creatine supplementation have shown promising results for counteracting at least some of the performance deficits caused by acute sleep loss [25, 26]. Blood creatinine levels are generally lower in those with obesity and T2D [27], and lower levels are associated with worse metabolic control. Whether creatine supplementation can counteract cognitive deficits following acute or chronic sleep loss, and whether its benefit differs by body-weight status, remains to be established.

Another metabolite pathway with roles that intersect metabolism and central nervous system function is the tryptophan–kynurenine axis. Tryptophan increased after TSD in our participants with obesity but not those with normal weight. A prior one-night TSD study by Davies et al. [8], reported that tryptophan levels increased in normal-weight-to-overweight men (BMI ~24.9 kg/m^2^), suggesting that the directional effect of TSD on tryptophan may not be specific to body-weight status, but may depend on cohort characteristics. The increase in tryptophan levels may be consistent with – but does not establish – a role in the well-described, transient antidepressant effect of total sleep deprivation [28, 29]. Downstream along this pathway, kynurenine levels decreased after TSD only in the normal-weight individuals. People with obesity may exhibit lower circulating levels of tryptophan as well as higher levels of kynurenine compared with controls without obesity [30, 31], whereas individuals with depression – a condition linked to sleep disruption – exhibit lower levels of kynurenine according to some analyses [32]. Peripherally, kynurenine exerts several cardiometabolic effects including vasodilation during inflammation [33]; kynurenine can also enter into the brain, where it can be converted to kynurenic acid (KYNA). In rats, KYNA has been found to reduce REM sleep and increase wake duration [34]. Although the two groups in our study exhibited significant responses for different tryptophan pathway metabolites, the directionality across the pathway converged: the kynurenine-to-tryptophan ratio decreased after TSD compared with NS in both groups. This ratio is widely used as a peripheral proxy for the activity of indoleamine 2,3-dioxygenase (IDO), and the shared decrease may reflect an acute relative shift in the circadian regulation of the pathway, consistent with our prior work showing that one night of sleep loss induces tissue-specific changes in clock-regulated metabolism [7]. Single-timepoint peripheral sampling cannot resolve whether the ratio change reflects altered enzymatic flux, circadian phase shifts, or for example redistribution of KYNA to the brain. Further studies are warranted to examine if altered peripheral and/or central kynurenine metabolism may also be involved in cardiometabolic effects of TSD in humans, and if such changes differ depending on body weight status.

## Strengths and limitations

The principal strength of this study is the randomized within-subject crossover design. This controls for inter-individual variability in baseline metabolite levels that can be altered in obesity. Furthermore, we used polysomnography to verify sleep during the NS condition and enable a comparison between the body weight conditions. However, there are several limitations to our present study. First, our sample size was based on similar published studies that have examined within-subject changes (refs [8, 18]) and was not formally powered to detect interaction effects (sleep condition × weight group, or condition × sex). Our findings should therefore be considered exploratory and hypothesis-generating, and whether they represent genuinely body-weight-specific or sex-specific responses requires validation in larger cohorts. Our study was furthermore not powered to detect between-group differences in objective sleep parameters, which studies indicate may require larger cohorts [35]. Second, we were only able to identify a targeted set of 55 metabolites. While these were selected for analytical robustness and run against authentic standards, the limited metabolite set means we could not assess several pathways implicated in sleep loss (such as a broader set of lipid species), and this also limited our ability to perform pathway enrichment analyses. Third, our study only comprised a single night of sleep loss. It is therefore not known whether body weight status also can modulate the effect of recurrent partial sleep restriction, or from recurrent shift work. Fourth, all female participants were studied during their active phase of combined hormonal contraceptive treatment. While this was done to control for cycle-related variability, it limits generalization to non-users. Finally, while we focused on within-subject and within-group metabolite changes, we were unable to control any potential degree of sleep-disordered breathing, and as such, sleep apnea may have confounded the response in the group with obesity.

## Conclusion

By examining within-individual metabolomic responses to total sleep deprivation in adults with and without obesity, our study extends prior work on metabolomics in the context of sleep loss – work that has predominantly been conducted in normal-weight or mixed-BMI cohorts. Our findings indicate that acute sleep loss produces both shared and group-specific metabolic responses: acylcarnitine and tryptophan–kynurenine pathway responses were directionally consistent across body-weight groups, whereas histidine and creatinine responses differed between groups. Together, these results suggest that body-weight status may shape the metabolic response to acute sleep loss. Whether recurrent bouts of sleep loss-induced disruption in these pathways can exacerbate metabolic dysfunction in those with obesity remains to be determined.

## Electronic supplementary material

Below is the link to the electronic supplementary material.

Supplementary Material 1

## Data Availability

Anonymized data and software code underlying this article can be shared by the corresponding author on reasonable request, to qualified researchers from accredited academic institutions.

## Abbreviations

KTR: Kynurenine-to-tryptophan ratio
NS: Normal sleep
REM: Rapid eye movement
TSD: Total sleep deprivation
