# Supplementary material for: Body-weight-specific and shared metabolomic responses to acute sleep loss in young adults
Source: J Transl Med. 2026 Jun 3;24:766. doi: 10.1186/s12967-026-08350-4 (PMC13263921; doi:10.1186/s12967-026-08350-4)
Supplement: Supplementary file 1 — Supplementary Material 1 [file 12967_2026_8350_MOESM1_ESM.docx]

**Supplementary Figure legends**

**A**


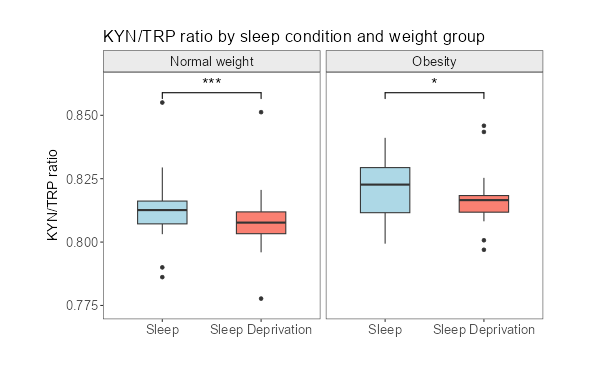


**B**


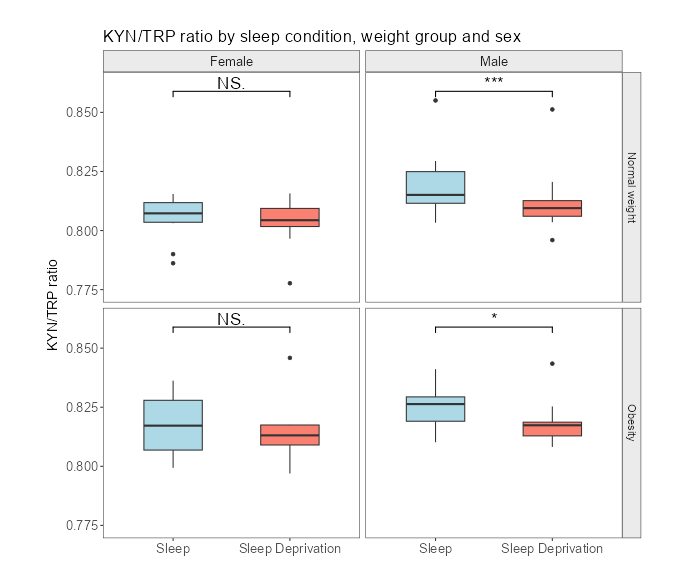


***Fig. S1)*** ***Effect of total sleep deprivation (TSD) compared with a full night of normal sleep (NS) on the kynurenine-to-tryptophan ratio.*** A) Analysis within the adults with normal body weight (left) and in the adults with obesity; B) Each body weight group further divided by female and male sex. For each comparison, analyzed as the within-subject ratio of kynurenine (KYN) to tryptophan (TRP), with statistical comparisons between sleep conditions using the Wilcoxon signed rank test (*, P < 0.05; ***, P < 0.001). Normal weight: n=24; Obesity: n=18.
